# Design and Development of a Triple-Band Multiple-Input–Multiple-Output Antenna for Sensing Applications

**DOI:** 10.3390/mi13122240

**Published:** 2022-12-16

**Authors:** Dinesh Kumar Raheja, Sachin Kumar, Shubhro Chakrabartty, Binod Kumar Kanaujia

**Affiliations:** 1 Department of Electronics and Communication Engineering, Netaji Subhas University of Technology (East Campus), Delhi 110031, India; 2 Department of Electronics and Communication Engineering, SRM Institute of Science and Technology, Kattankulathur 603203, India; 3 Department of Electronics and Communication Engineering, K L University, Vijayawada 522302, India; 4 School of Computational and Integrative Sciences, Jawaharlal Nehru University, New Delhi 110067, India

**Keywords:** circular polarization, elliptical, MIMO, multiband, stacking

## Abstract

In this article, a triple-band quad-element stacked multiple-input–multiple-output (MIMO) antenna is proposed for sensing applications. Each radiating element of the presented MIMO antenna consists of a diagonally truncated square patch, which is proximity coupled to the elliptical radiating patch. The proposed MIMO antenna is designed to resonate for three frequencies (4.2, 4.8, and 5.8 GHz) in the C-band range. The antenna shows circular polarization characteristics at 4.2 and 4.8 GHz frequencies. Each stacked element of the proposed antenna is excited independently through a 50 Ω coaxial feed. The Rogers RT Duroid/5880 dielectric substrate is used for the fabrication of two layers of the stacked MIMO antenna. The presented stacked MIMO antenna simulation and experimental outcomes are in good agreement.

## 1. Introduction

Multiple-input–multiple-output (MIMO) antenna-based communication is a preferred area of research for industry and academia, as it can overcome challenges faced due to multiple reflections and diffractions from the edges of large buildings and irregular objects [1]. The MIMO system design approach becomes more challenging for modern mobile communications, including civil, military and emergency services, due to highly unpredictable dynamics of ambience around the terminal users. To minimize the adverse effects of multipath fading signals, the technique of antenna diversity is preferred for both commercial and personal applications [2]. Considering the high data-rate transfer requirements of modern digital communication systems, large bandwidth and more channels are considered favorably, though both are difficult to increase and lay a major challenge for communication engineers. Therefore, in such a situation, the concept of MIMO is applied by using spatial diversity and multiplexing. Various narrowband, broadband and ultra-wideband (UWB) based MIMO systems for radar, cognitive radio, long-term evolution (LTE), mobile data communication, wireless local area network (WLAN), and worldwide interoperability for microwave access (Wi-MAX) have already been designed and reported [3,4,5,6,7,8]. Several wideband antenna elements configured parallel or orthogonally, supported with a partial ground or defected ground structures (DGS), have been developed [9,10,11]. Many of them deploy selective band-notch filtering techniques since wideband systems are susceptible to severe interferences, as they partially share the same channel bandwidths with several neighboring users [12]. Other design approaches for narrowband, dual-band, and broadband MIMO systems deploying spatial, frequency, or polarization diversity have also been reported [13,14].

MIMO antenna designing becomes more challenging for navigation, mobile systems, aviation, and military applications primarily due to the non-availability of enough space. In the MIMO structure, at least two similar antenna elements have to be inevitably accommodated in very close proximity. This increases mutual coupling and the envelope correlation coefficient (ECC) amongst the resonant elements of the MIMO antenna. If the spacing between two antenna elements is insufficient, the interference rises to a practically unacceptable level, and the performance of the MIMO antenna gets deteriorated. Therefore, orthogonal arrangement of the antenna elements with polarization diversity is preferred in many designs [15]. Further, if the MIMO system has more than two antenna elements, the complications associated with mutual coupling and ECC become worse, and the antenna design becomes even more critical [16,17]. Additionally, in the reported MIMO configurations, the ground patches of the antenna unit cells are not linked to each other, thus having a varying reference voltage [18].

The objective of this paper is to design a triple-band quad-element MIMO antenna with connected ground planes. The basic unit of the MIMO antenna is a stacked configuration of the elliptical patch and square patch radiators, here treated as a single antenna element. In the single antenna element, the two patches are centrally aligned and symmetrically placed around the diagonals. Starting with a single antenna element, 1 × 2 (two elements) and 2 × 2 (four elements) MIMO antennas are developed with connected ground planes to operate at 4.2, 4.8, and 5.8 GHz, in the C-band, with good isolation level and very low ECC. Moreover, the proposed antennas exhibit circular polarization at 4.2 and 4.8 GHz. The proposed MIMO antenna is simple to fabricate, easily integrable, low profile, and compact in size. For optimal performance, the dimensions of single, dual, and quad-port MIMO antennas are optimized by using the finite-element-method-based ANSYS HFSS simulator.

## 2. Antenna Design

### 2.1. Stacked Antenna Element

**Figure 1:**
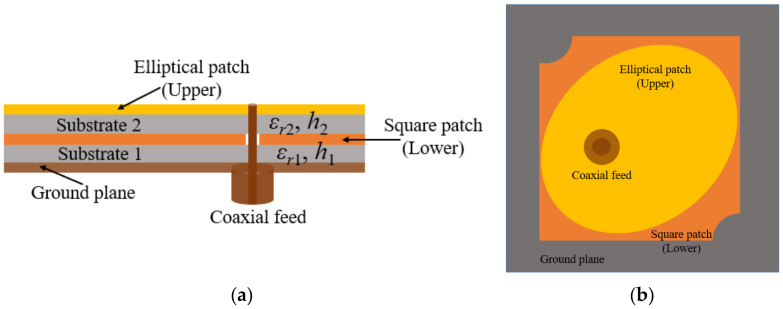
Schematic of the stacked antenna element (**a**) side view (**b**) top view.

Figure 1 represents the schematic of the stacked antenna element. The proposed stacked antenna consists of a diagonally truncated square patch (present in the middle between the two substrates (1 and 2) and represented in orange color) that is proximity coupled to the elliptical radiating patch (present at the top and represented through yellow color). On the bottom of the antenna, a ground plane, depicted in brown, is present. The lower and upper substrate layers (1 and 2) are shown in grey. The resonating frequency of the lower patch is calculated as

(1) $$f_{1}=\frac{\alpha c}{2\pi r_{eff}\sqrt{\varepsilon_{r,\;eff}}}$$

where c is the speed of light in vacuum, and α is the first zero of the derivative of Bessel function of the first order.

The effective dielectric constant is calculated by [19].

(2) $$\begin{array}{c} \varepsilon_{r,\;eff}=\varepsilon_{r1}p_{1}+\varepsilon_{r1}{\left(1-p_{1}\right)}^{2}\times \left[\varepsilon_{r2}{}^{2}p_{2}p_{3}+\varepsilon_{r2}\left\{p_{2}p_{4}+{\left(p_{3}+p_{4}\right)}^{2}\right\}\right]\\ \times {\left[\varepsilon_{r2}{}^{2}p_{2}p_{3}p_{4}+\varepsilon_{r1}\left(\varepsilon_{r2}p_{3}+p_{4}\right){\left(1-p_{1}-p_{4}\right)}^{2}+\varepsilon_{r2}p_{4}\left\{p_{2}p_{4}+{\left(p_{3}+p_{4}\right)}^{2}\right\}\right]}^{-1} \end{array}$$

where $\varepsilon_{r1}$ is the dielectric constant of the lower substrate and $\varepsilon_{r2}$ is the dielectric constant of the superstrate. The different parameters of Equation (1) are calculated as [19]

(3) $$p_{1}=1-\frac{h_{1}}{2w_{e}}\ln\left(\frac{\pi w_{e}}{h_{1}}-1\right)-p_{4}$$

(4) $$p_{2}=1-p_{1}-p_{3}-2p_{4}$$

(5) $$p_{3}=\frac{h_{1}-g}{2w_{e}}\ln\left[\frac{\pi w_{e}}{h_{1}}\frac{\cos\left(\frac{\pi g}{2h_{1}}\right)}{\pi \left(\frac{1}{2}+\frac{h_{2}}{h_{1}}\right)+\frac{g\pi }{2h_{1}}}+\sin\left(\frac{g\pi }{2h_{1}}\right)\right]$$

(6) $$p_{4}=\frac{h_{1}}{2w_{e}}\ln\left(\frac{\pi }{2}-\frac{h_{1}}{2w_{e}}\right)$$

(7) $$g=\frac{2h_{1}}{\pi }\arctan\left[\frac{\frac{\pi h_{2}}{h_{1}}}{\frac{\pi }{2}\left(\frac{w_{e}}{h_{1}}\right)-2}\right]$$

(8) $$w_{e}=\sqrt{\frac{\varepsilon_{r}^{\prime }}{\varepsilon_{r,\;eff}}}\left[\left\{w+0.882h_{1}+0.164h_{1}\left(\frac{\varepsilon_{r}^{\prime }-1}{\varepsilon_{r}^{\prime }{}^{2}}\right)\right\}+h_{1}\left(\frac{\varepsilon_{r}^{\prime }-1}{\pi \varepsilon_{r}^{\prime }}\right)\left\{\ln\left(0.94+\frac{w}{2h_{1}}\right)+1.451\right\}\right]$$

(9) $$\varepsilon_{r}^{\prime }=\frac{2\varepsilon_{r,\;eff}-1+{\left(1+\frac{10h_{1}}{w_{e}}\right)}^{-0.5}}{1+{\left(1+\frac{10h_{1}}{w_{e}}\right)}^{-0.5}}$$

(10) $$\varepsilon_{r}^{\prime }=\frac{2\varepsilon_{r,\;eff}-1+{\left(1+\frac{10h_{1}}{w_{e}}\right)}^{-0.5}}{1+{\left(1+\frac{10h_{1}}{w_{e}}\right)}^{-0.5}}$$

where $h_{1}$ and $h_{2}$ are the thicknesses of the lower and upper substrates, respectively, and r is the radius of the upper radiating patch. By using the relation of equivalence, Equation (10) can be evaluated [20]. The parameters $w_{e}$ and $\varepsilon_{r}^{\prime }$ are evaluated by means of the iteration method in [21]. The effective radius size is calculated by using the relation [22]

(11) $$r_{eff}=r{\left\{1+\frac{2h}{\pi \varepsilon_{re}r}\left[\log\left(\frac{r}{2h}\right)+1.41\varepsilon_{re}+1.77+\frac{h}{r}\left(0.268\varepsilon_{re}+1.65\right)\right]\right\}}^{0.5}$$

(12) $$\varepsilon_{re}=\frac{\varepsilon_{r1}h}{h_{2}+h_{1}\varepsilon_{r1}}$$

(13) $$h=h_{1}+h_{2}$$

**Figure 2:**
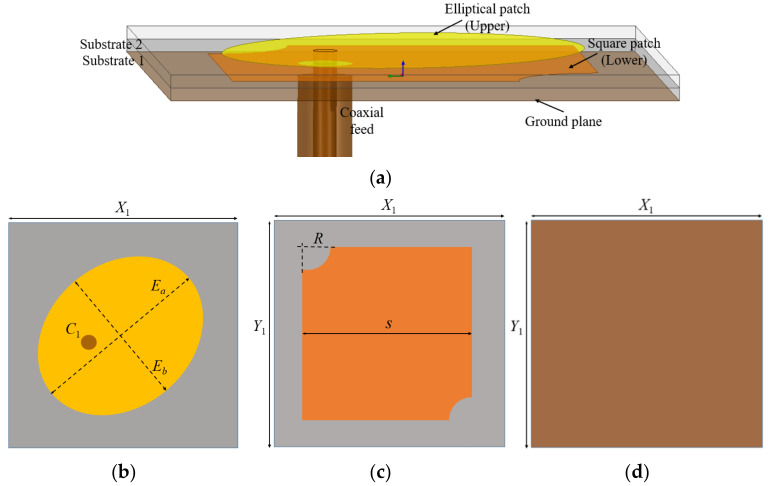
Geometry of the stacked antenna element: (**a**) side view; (**b**) elliptical patch (top layer); (**c**) square patch (middle layer); (**d**) ground plane (bottom layer).

Rogers RT Duroid/5880 substrate material with relative permittivity of 2.2 and thickness of 0.76 mm is used for designing the upper and lower layers of the stacked antenna element. Figure 2 represents the geometrical details of the stacked antenna element. The stacked antenna is comprised of an elliptical patch (on the top), truncated square patch (in the middle), and square-shaped ground surface in the bottom. An elliptical radiating patch with high eccentricity is placed on the top and directly fed by a 50 Ω coaxial feed. The major axis of the elliptical disk is aligned at 45° with reference to the *X*-axis and in order to obtain a dual band resonance in the elliptical patch, it is required to excite two resonating modes simultaneously. This is done by using a coaxial feed at 45° to both the major (*E_a_*) and minor axes (*E_b_*) of the elliptical patch, with proper impedance matching. Furthermore, the third resonant band is achieved by introducing proximity coupled parasitic square patch with two diagonally truncated corners just below the primary elliptical patch. As a result, three resonances are achieved from the stacked antenna configuration. The circular polarization is achieved with the help of an elliptical patch and diagonally truncated corners of the lower square patch. In Figure 2, *C*_1_ corresponds to the circle required for exciting the elliptical radiator using 50 Ω coaxial feed through SMA connector. The dimensions for designing the antenna elements are summarized in Table 1.

**Figure 3:**
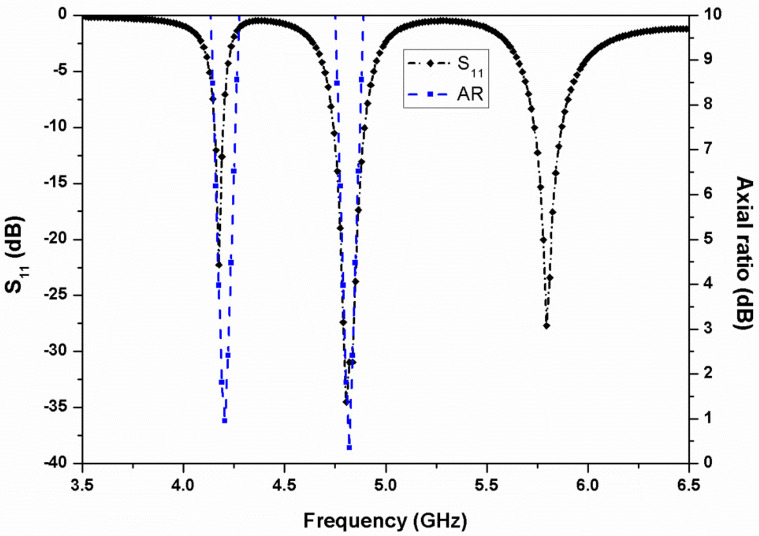
Simulated S_11_ and axial ratio of the stacked antenna element.

**Figure 4:**
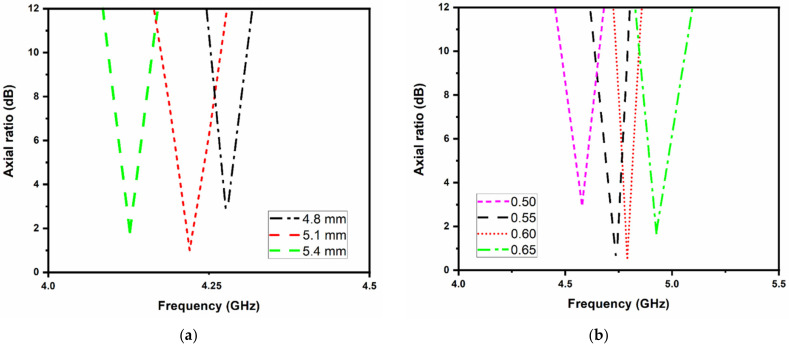
Effect of the antenna dimensions on axial ratio; (**a**) square patch truncation (*R*); (**b**) ellipticity (*c*).

The reflection coefficients and axial ratio characteristics of the two-layer stacked antenna are shown in Figure 3. It can be observed that the antenna resonates at 4.2, 4.8, and 5.8 GHz with circular polarization performance in the two lower frequency bands. For controlling the resonating bands, the elliptical patch and truncated square patch dimensions can be varied. It is also observed that by changing the dimensions of the corner truncation (of the square patch) and ellipticity of the radiating patch, the axial ratio bandwidth can be altered according to the application bands. The effect of square patch truncation (*R*) and ellipticity (*c*) on axial ratio is illustrated in Figure 4. When the truncation increases, the axial ratio degrades, as shown in Figure 4a, whereas a large ellipticity improves the axial ratio, as shown in Figure 4b.

### 2.2. Two-Port MIMO Antenna

**Figure 5:**
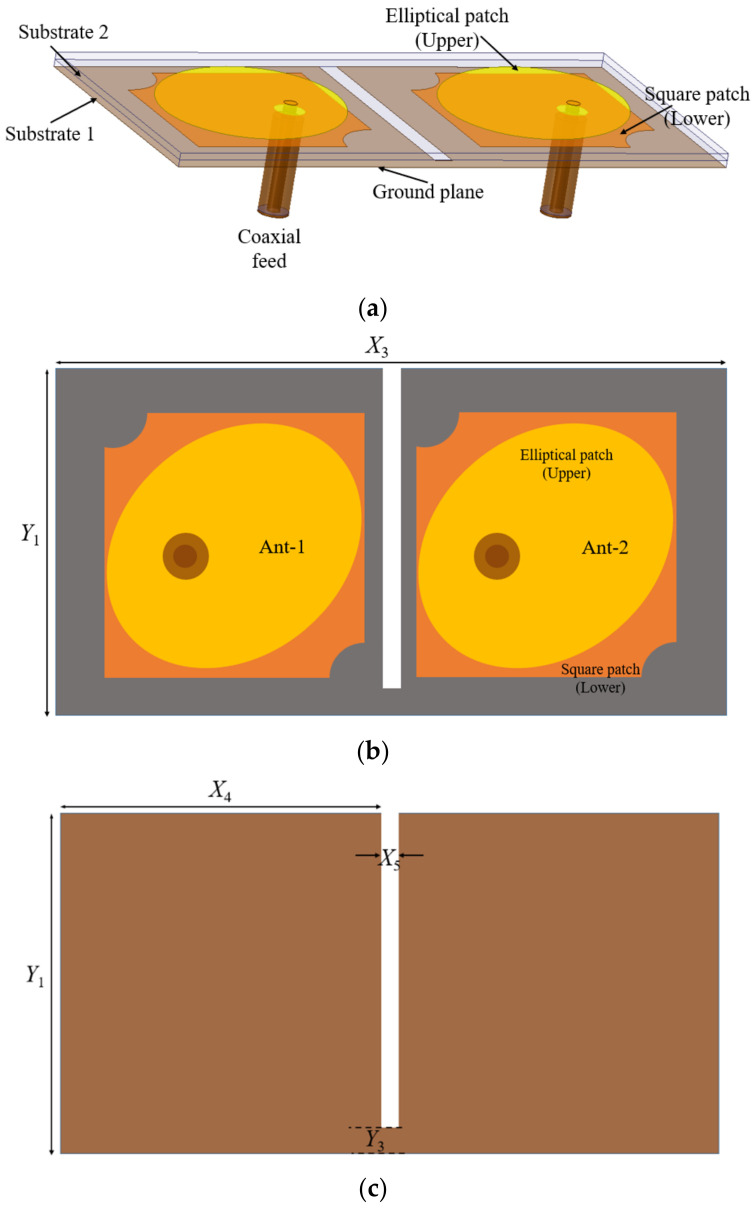
Geometry of the two-element stacked MIMO antenna: (**a**) side view; (**b**) top view; (**c**) ground plane.

While designing the two-element MIMO antenna, the principle of spatial diversity is followed. The schematic and geometrical details of the two-port stacked MIMO antenna are given in Figure 5. The two identical stacked antenna elements Ant-1 and Ant-2 are placed parallel and in close proximity to each other. The substrate RT Duroid/5880 of the thickness of 0.76 mm is used for both the layers of the MIMO antenna, as used in the reference stacked antenna element. The overall size of the two element MIMO antenna is *X*_3_ × *Y*_1_ mm^2^. The edge-to-edge gap between the two adjacent elements in the ground plane is kept ~0.028*λ*_0_ at 4.2 GHz. A rectangular slot (2 mm × 28 mm) is introduced into the ground plane conductor to keep mutual coupling sufficiently below the acceptable limits.

**Figure 6:**
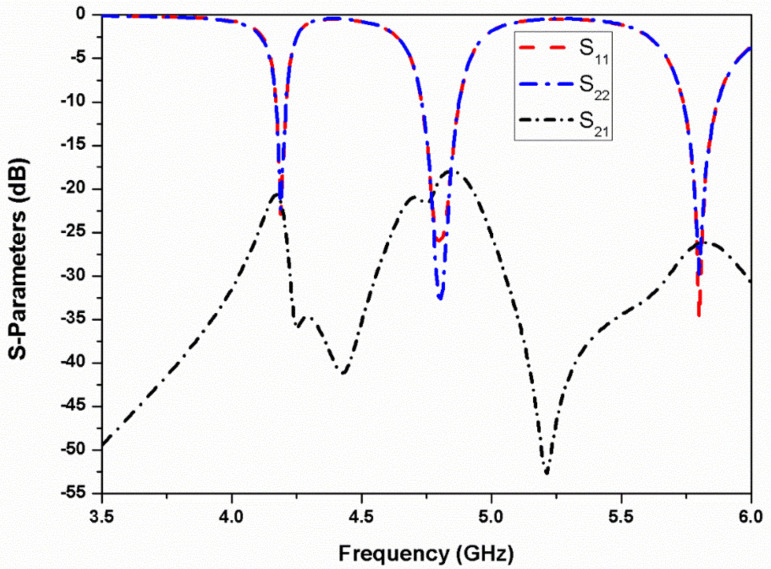
Simulated S-parameters of the two-element stacked MIMO antenna.

**Figure 7:**
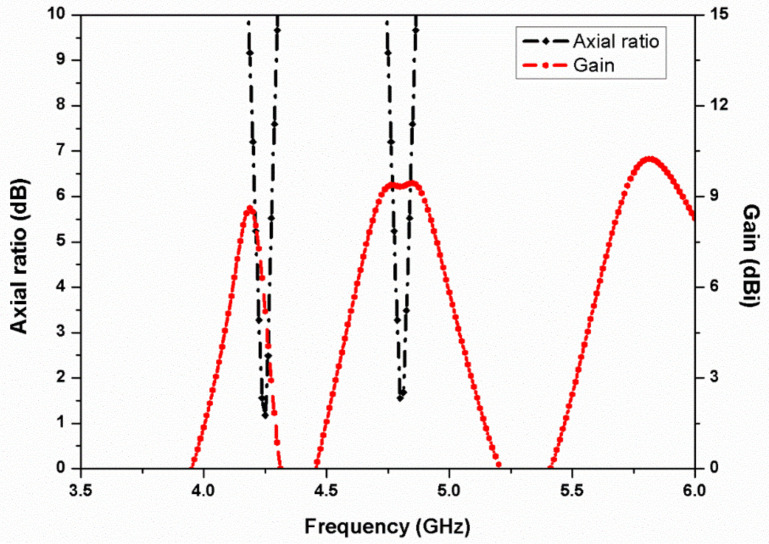
Simulated axial ratio and gain of the two-element stacked MIMO antenna.

Figure 6 and Figure 7 represent the reflection coefficients, isolation, axial ratio, and gain characteristics of the proposed stacked two-port MIMO antenna. From Figure 6, it can be observed that the antenna is resonating for three frequency bands (4.2, 4.8, and 5.8 GHz) with isolation of more than 18 dB at all the resonating bands. When the two stacked antenna elements were placed in close proximity, the isolation level was degraded due to the effect of mutual coupling since both the antennas are operating for the same frequency bands. The isolation level was not more than 13 dB and due to this reason, a rectangular slot was introduced in the ground surface, which reduced the mutual coupling effect between the two radiating elements. Additionally, the two-port antenna shows circular polarization at 4.2 and 4.8 GHz, and the peak gain of the MIMO antenna ranges from 5.5 to 7 dBi at the resonating frequency bands as illustrated in Figure 7.

**Figure 8:**
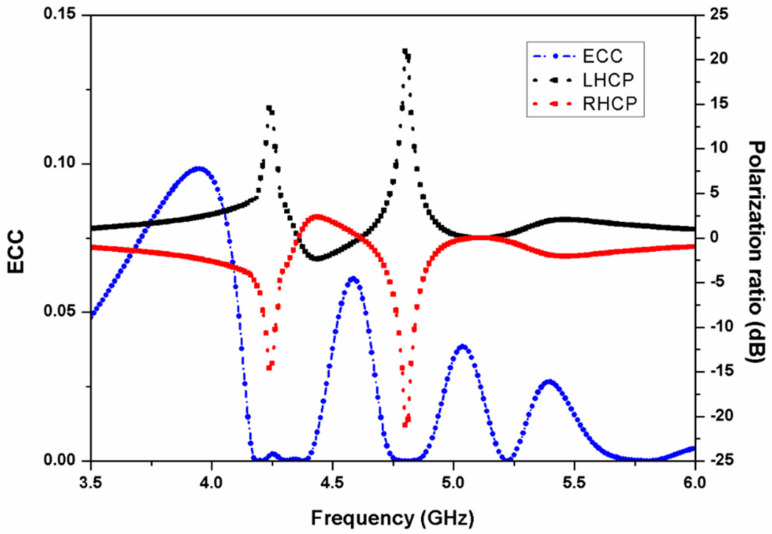
ECC and polarization ratio response of the two-element stacked MIMO antenna.

Figure 8 represents the ECC and polarization ratio response of the two-element stacked MIMO antenna. In MIMO antennas, the mutual coupling between the two neighboring ports can be studied by computing ECC. The value of ECC can be calculated by using the relation [23]

(14) $$\rho_{eij}=\frac{{\left|S_{ii}^{*}S_{ij}+S_{ji}^{*}S_{jj}\right|}^{2}}{\left(1-{\left|S_{ii}\right|}^{2}-{\left|S_{ji}\right|}^{2}\right)\left(1-{\left|S_{jj}\right|}^{2}-{\left|S_{ij}\right|}^{2}\right)}$$

The curve in Figure 8 shows that the value of ECC is negligibly small at the resonant bands, thus illustrating diversity performance of the presented two-port MIMO antenna. Further, from the polarization ratio graph, it can be concluded that the proposed antenna radiates left-hand circularly polarized (LHCP) waves in the two frequency bands.

### 2.3. Four-Port MIMO Antenna

**Figure 9:**
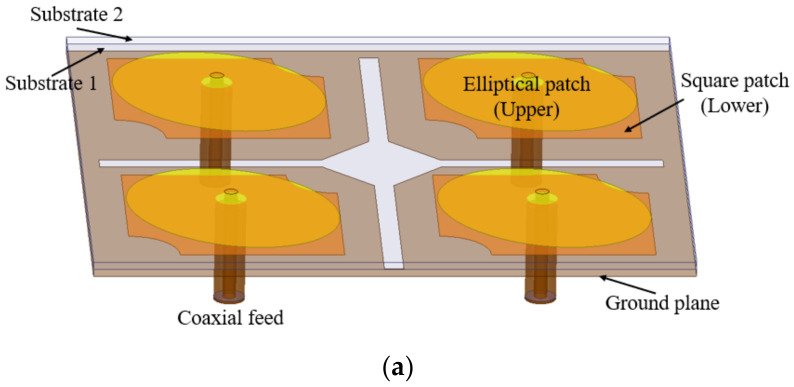

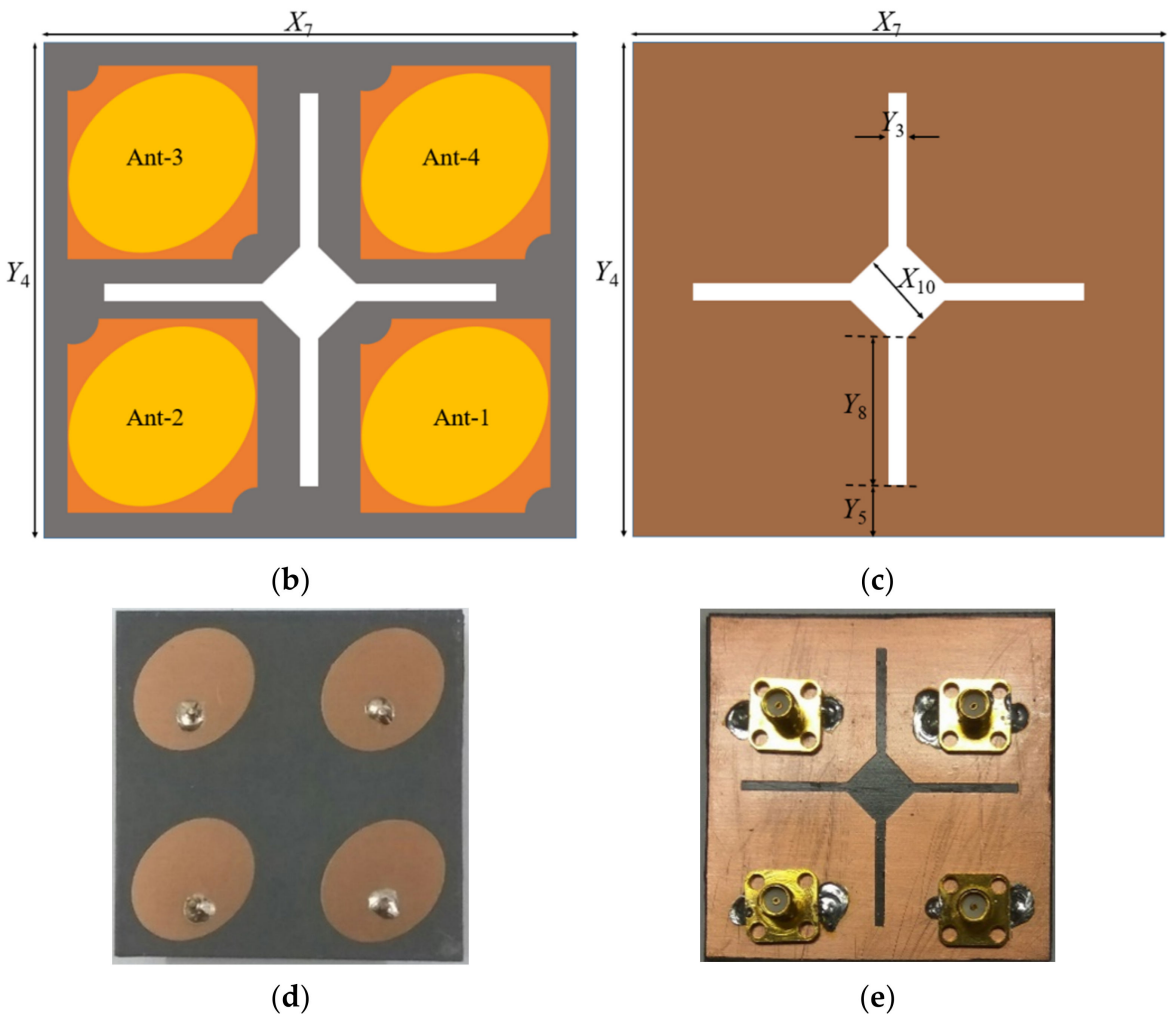
Geometry of the four-element stacked MIMO antenna: (**a**) side view; (**b**) top view; (**c**) ground plane; (**d**) antenna prototype (top view); (**e**) antenna prototype (bottom view).

The four-element MIMO antenna is designed using the same stacked antenna element configured in two rows and two columns along the *X*- and *Y*-axis as illustrated in Figure 9a–c. The total size of the designed four-port MIMO antenna is *X*_7_ × *Y*_4_ mm^2^. Since the four-port MIMO antenna assembly contains closely packed identical unit cells placed in 2 × 2 configuration, the mutual coupling and cross-correlation levels are apparently much higher in comparison to the two element MIMO antenna. So, the overall performance of the four-element MIMO antenna may be severely affected, and, therefore isolation amongst all the resonating elements needs to be increased. Considering the overall size of the MIMO antenna, there is a limitation on the size of isolation slots in the ground plane, due to space constraints. However, the inter-element isolation can be further improved by increasing the diagonal separation amongst all the antenna elements. Therefore, a diagonally aligned square slot (*X*_10_ × *X*_10_ mm^2^) is also etched in the ground plane conductor along with the four symmetrical rectangular slots. The stacked MIMO antenna prototype top and bottom views are shown in Figure 9d,e, respectively.

## 3. Results

**Figure 10:**
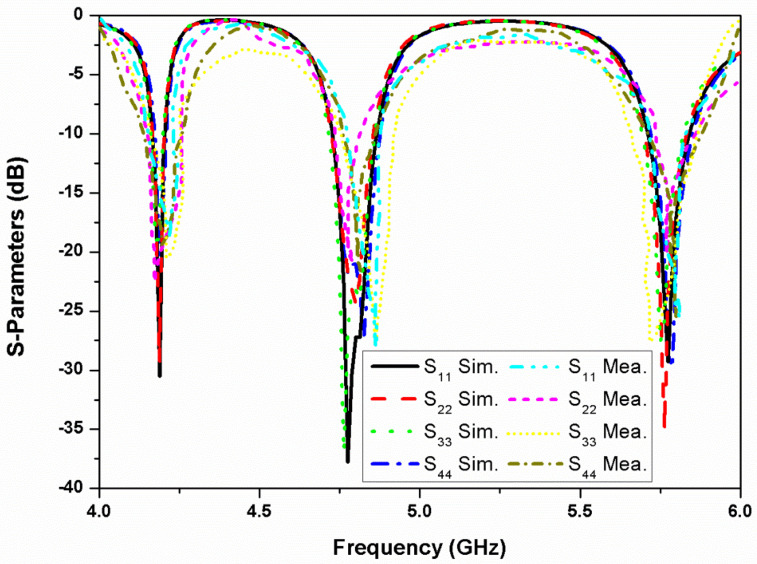
Simulated and measured reflection coefficients of the four-element stacked MIMO antenna.

The MIMO antenna performance is measured by using the Agilent vector network analyzer of PNA-L series. Figure 10 represents simulated and measured reflection coefficient characteristics of the four antenna elements. In the course of S-parameters measurement at port one, the other ports in the stacked configuration are terminated using 50 Ω load. The antenna exhibits (S_11_ < −10 dB) impedance bandwidth of 4.15–4.25 GHz (2.4%) for the first band, 4.7–4.85 GHz (3.1%) for the second band, and 5.7–5.85 GHz (2.6%) for the third resonating band. However, by varying the side length of the proximity-coupled square patch, the resonating frequencies can be controlled. Similarly, by altering the ellipticity of the primary radiating patch, the frequency can be varied.

**Figure 11:**
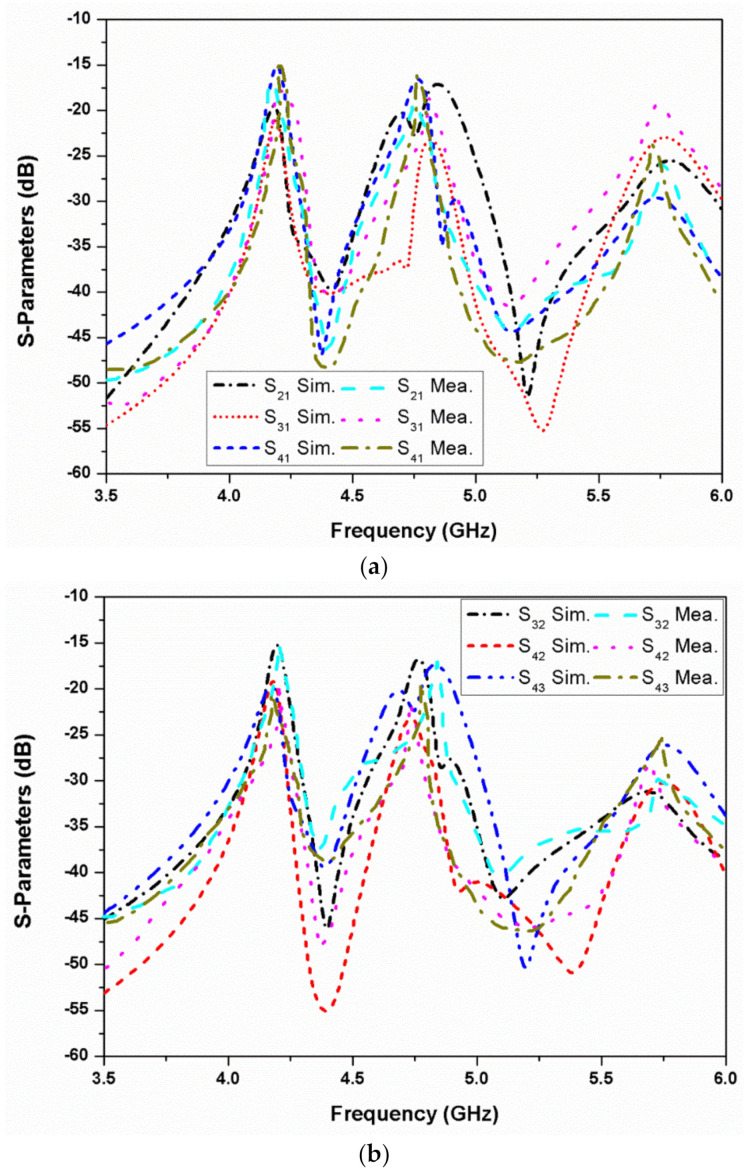
Simulated and measured S-parameters of the four-element stacked MIMO antenna: (**a**) when port 1 is excited; (**b**) when port 2/port 3 is excited.

**Figure 12:**
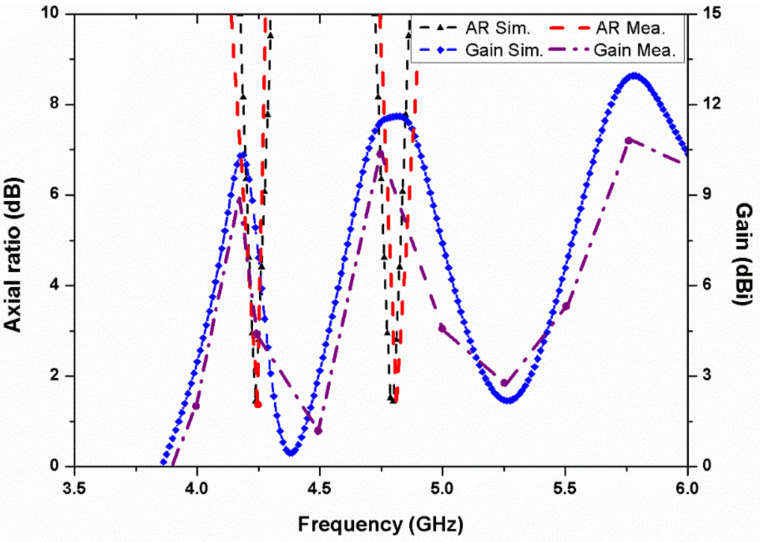
Simulated and measured axial ratio and gain of the four-element stacked MIMO antenna.

Figure 11 represents the simulated and measured mutual coupling between different ports of the proposed four-element stacked MIMO antenna. As can be seen, the isolation achieved is greater than 15 dB in the first band, more than 18 dB in the second band, and greater than 20 dB for the third resonance. Hence, good isolation is realized in both the simulated and measured results for all four ports. The antenna simulated and measured axial ratio and gain behavior are represented in Figure 12. The stacked MIMO antenna shows circular polarization at 4.2 and 4.8 GHz, and the measured peak gain achieved is 7 dBi at the 5.8 GHz resonance. The measured outcomes of the fabricated stacked MIMO antenna are in agreement with the simulated outcomes. A minor variation in results is seen, which is due to the customary fabrication process and cable effect. It may also be due to the alignment of the stacked patches and conductive adhesives used during fabrication. Other losses, such as conductor, surface radiation, and power leakage from the SMA connector soldering, are also present in the measurements, which were almost negligible in the simulated results. The value of ECC for a four-port MIMO system is calculated as [24]

(15) $$\begin{array}{l} \rho \left(i=1,\;j=2,\;4\right)\\ =\frac{{\left|S_{11}^{*}S_{12}+S_{21}^{*}S_{22}+S_{13}^{*}S_{32}+S_{14}^{*}S_{42}\right|}^{2}}{\left(1-{\left|S_{11}\right|}^{2}-{\left|S_{21}\right|}^{2}-{\left|S_{31}\right|}^{2}-{\left|S_{41}\right|}^{2}\right)\left(1-{\left|S_{12}\right|}^{2}-{\left|S_{22}\right|}^{2}-{\left|S_{32}\right|}^{2}-{\left|S_{42}\right|}^{2}\right)} \end{array}$$

**Figure 13:**
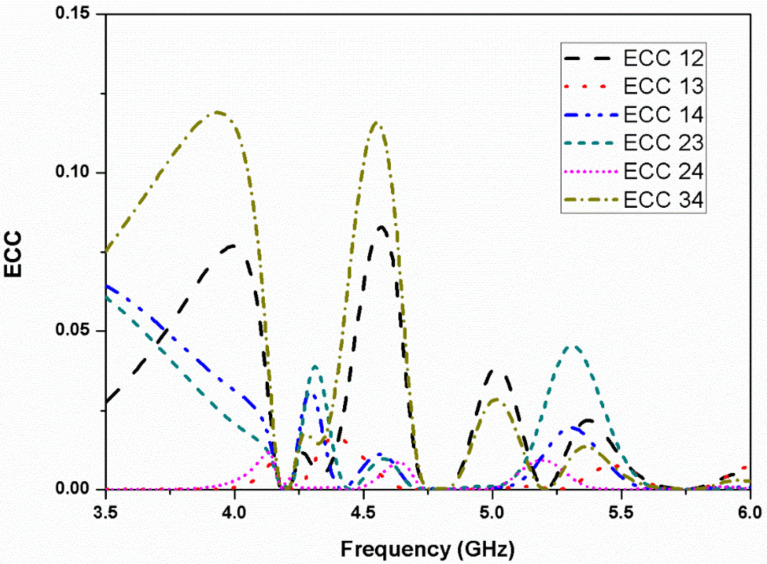
ECC of the four-element stacked MIMO antenna.

**Figure 14:**
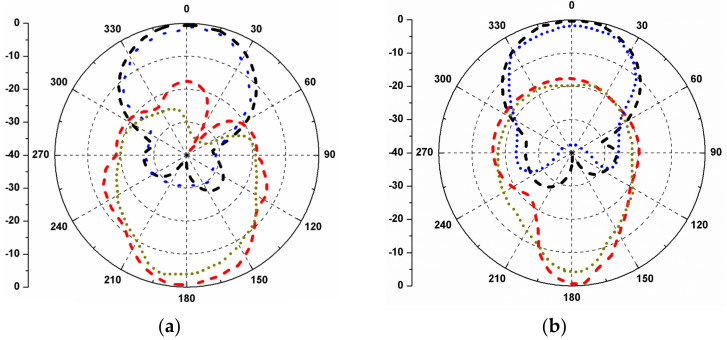

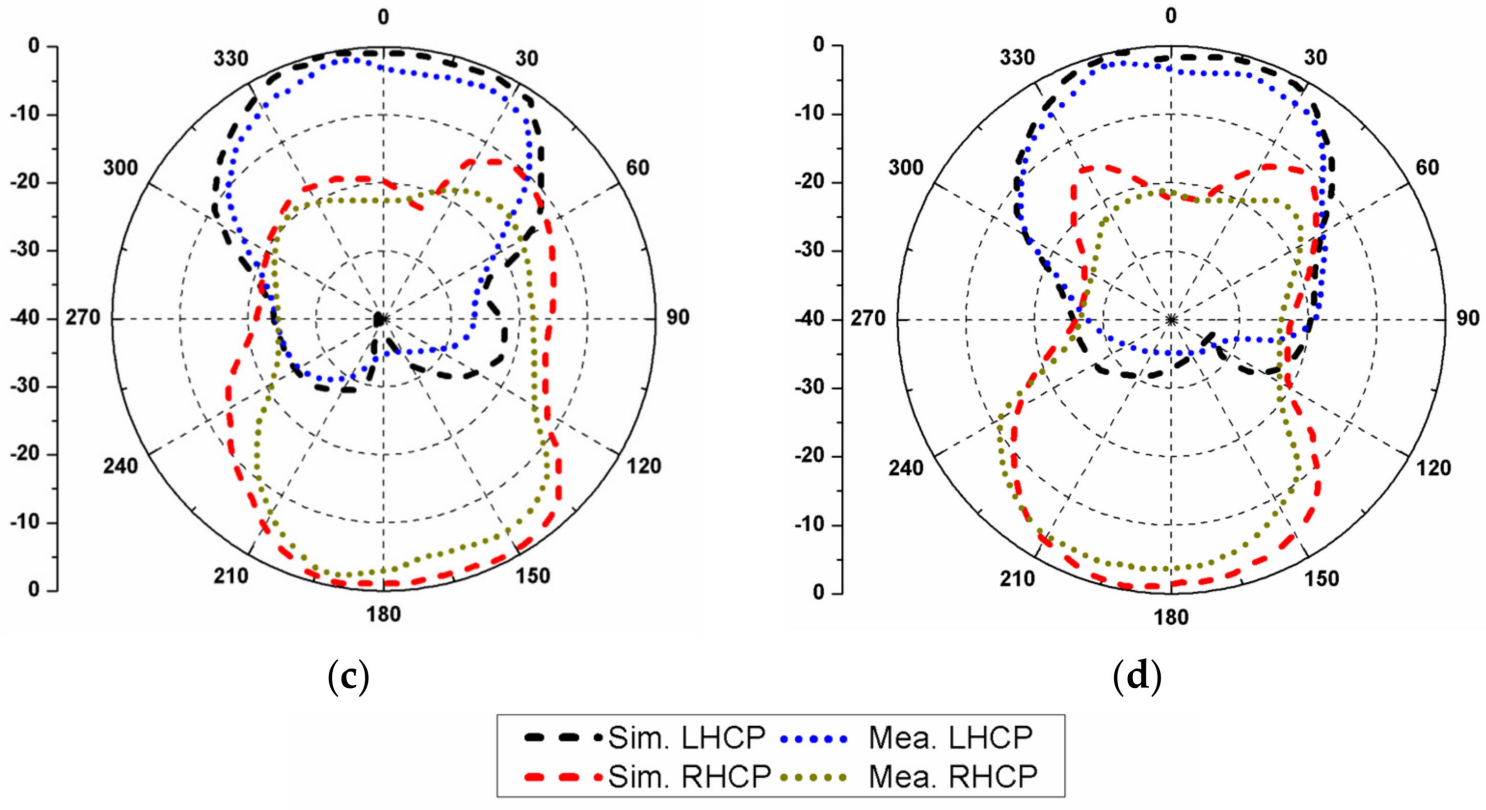
Simulated and measured radiation patterns of the stacked MIMO antenna: (**a**) *XZ*-plane/4.2 GHz; (**b**) *YZ*-plane/4.2 GHz; (**c**) *XZ*-plane/4.8 GHz; (**d**) *YZ*-plane/4.8 GHz.

The ECC of the proposed four element stacked MIMO antenna is negligibly small and is plotted in Figure 13. The ECC values are less than 0.05 at the three resonating bands, which assures that the MIMO antenna exhibits good diversity performance. Furthermore, the antenna diversity gain (DG) is evaluated, and it is noticed that the value of DG is greater than 9.9 dB, as can be seen in Table 2. The radiation patterns of the proposed stacked antenna at 4.2 and 4.8 GHz are shown in Figure 14. It can be seen that the antenna radiates LHCP waves in the +*Z*-direction and right-hand circularly polarized (RHCP) waves in the –*Z*-direction when port-1 is excited and other ports are matched.

## 4. Performance Comparison

Table 3 shows the comparison of the stacked MIMO antenna with the reported multiband MIMO antennas. The antenna structures shown in [25,26] were larger in size, and had a high design complexity, dual bands with linear polarization, and only two resonators. The triple band MIMO antenna structures described in [27,28] were four-element configurations with relatively large size and linear polarization behavior. The MIMO antenna proposed in [29] was made up of four resonators, but it had a low gain and linear polarization behavior. Additionally, the ground planes of the antennas had some defects to improve isolation. The proposed antenna, on the other hand, has a stacked geometry with four elements that provides triple-band resonances and a whole ground plane. In addition, circular polarization is obtained in two bands, and the antenna gain is high despite its small size. The features of the proposed antenna are as follows:

- In contrast to previous antenna designs [25,26,27,28,29], the MIMO antenna employs stacked geometry to achieve multiple resonances.
- The proposed MIMO antenna has a straightforward design with elliptical and square patches, making fabrication easier, and it provides both linearly and circularly polarized operation.
- There is no decoupling element used in the proposed MIMO antenna, which contributes to its design simplicity.
- Unlike other works reported in the literature [25,26,27,28,29], the proposed antenna is fabricated on a full ground plane.
- The MIMO antenna is relatively small in size, consisting of four radiators, and has high diversity performance metrics.

## 5. Conclusions

The paper presents a triple-band quad-port stacked MIMO antenna with two circularly polarized bands (4.2 and 4.8 GHz) and connected ground planes. The presented MIMO antenna is composed of four identical stacked antenna elements excited by the help of 50 Ω coaxial feed using SMA connectors. For introducing circular polarization characteristics in the two bands, an elliptical-shaped primary resonator and truncated square patch secondary resonator excited through electromagnetic coupling are used. The peak gain of the designed MIMO antenna ranges from 5.5 to 7 dBi in the radiating bands. The proposed antenna can be a potential candidate for tracking and sensing applications, as it is circularly polarized and has a high gain. Additionally, by making some changes to its geometry, it can be used for RFID sensing applications in readers.

## Figures and Tables

**Table 1:** Stacked antenna design specifications and dimensions.

Antenna Specifications	Value	Antenna Specifications	Value
Substrate	Rogers RT Duroid/5880	*ε*_*r*1_/*ε*_*r*2_	2.2
Substrate thickness, *h*_1_/*h*_2_	0.76 mm	Ellipticity, *c*	60%
Major axis, *E_a_*	24 mm	Minor axis, *E_b_*	19.2 mm
Side of square patch, *S*	22 mm	Radius of truncation, *R*	5.1 mm
*X*_1_	30 mm	*Y*_1_	30 mm
*X*_3_	62 mm	*X*_4_	30 mm
*X*_5_	2 mm	*Y*_3_	2 mm
*X*_7_	62 mm	*Y*_4_	62 mm
*Y*_5_	5.5 mm	*X*_10_	10 mm
*Y*_8_	23 mm		

**Table 2:** Parameters of the proposed MIMO antenna.

Frequency (GHz)	Isolation (dB)	ECC	DG
4.2	>15	<0.02	10
4.8	>18	<0.02	9.98
5.8	>20	<0.02	10

**Table 3:** Performance comparison with the reported multiband MIMO antennas.

Ref.	Design Methodology	No. of Resonators	Polarization	(S_11_ ≤ −10 dB) Bands (GHz)	3-dB Axial Ratio Bands (GHz)	Antenna Size (mm^2^)	Connected Grounds	Isolation (dB)	ECC	Peak Gain (dB)	Design Complexity
[25]	Orthogonal arrangement	2	Linear	2.45, 3.5	---	132.8 × 70	Yes	>30	<0.15	5.9	Moderate
[26]	Primary and secondary reflectors	2	Linear	3.7, 4.1	---	60.6 × 48.5	Yes	>25	<0.01	8.5	High
[27]	Half-mode substrate integrated waveguide	4	Linear	4.43, 5.39	---	82 × 82	No	>35	<0.0025	6.4	High
[28]	Dipole with a feeding network	4	Linear	2.45, 3.2, 3.8	---	70 × 70	Yes	>20	<0.14	6.8	Moderate
[29]	Symmetrically oriented	4	Linear	3.77, 4.7, 6.31	---	32 × 32	Yes	>16	<0.1	3.3	Low
This work	Stacking	4	Linear + Circular	4.2, 4.8, 5.8	4.2, 4.8	62 × 62	Yes	>16	<0.12	7	Low

## Data Availability

The data presented in this study are available on request from the corresponding author.
